# Do nursing staff encourage functional activity among nursing home residents? A cross-sectional study of nursing staff perceived behaviors and associated factors

**DOI:** 10.1186/s12877-017-0412-9

**Published:** 2017-01-14

**Authors:** Nienke O. Kuk, Mirre den Ouden, G. A. Rixt Zijlstra, Jan P. H. Hamers, Gertrudis I. J. M. Kempen, Gerrie J. J. W. Bours

**Affiliations:** 1 Department of Health Services Research, CAPHRI Care and Public Health Research Institute, Maastricht University, P.O. Box 616, Maastricht, MD 6200 The Netherlands; 2 Research Centre for Autonomy and Participation of People with a Chronic Illness, Zuyd University of Applied Sciences, Faculty of Health, Heerlen, The Netherlands

**Keywords:** Activities of daily living, Contextual factors, Functional activity, Information-seeking behaviors, Nursing home, Nursing staff, Professional characteristics

## Abstract

**Background:**

Nursing home residents are mainly inactive. Nursing staff can encourage residents to perform functional activities during daily care activities. This study examines 1) the extent to which nursing staff perceive that they encourage functional activity in nursing home residents and 2) the associations between these nursing behaviors and professional characteristics, contextual factors, and information-seeking behaviors.

**Methods:**

In this cross-sectional study, 368 registered nurses and certified nurse assistants, working in somatic and psychogeriatric wards of forty-one nursing homes throughout the Netherlands participated. Self-reported data were collected with a questionnaire, comprising the MAINtAIN-behaviors, which assesses the extent to which nursing staff encourage functional activities, including different activities of daily living (ADL), household activities, and miscellaneous encouraging activities (e.g., discouraging informal caregivers from taking over activities residents can do themselves). Additional data collected included professional characteristics (e.g., age), contextual factors (e.g., ward type), and information-seeking behaviors (e.g., reading professional journals). Descriptive statistics were used to determine the extent to which functional activities were encouraged. Hierarchical linear regression analyses were performed to determine the associations between the encouragement of functional activities and other factors.

**Results:**

Nursing staff perceived that household activities (mean 4.1 (scale range 1–9), SD 1.9) were less often encouraged than ADL (mean 6.9, SD 1.2) or miscellaneous activities (mean 6.7, SD 1.5). The percentage of nursing staff stating that different household activities, ADL, or miscellaneous activities were almost always encouraged ranged from 11 to 45%, 41 to 86%, and 50 to 83% per activity, respectively. The extent to which these activities were encouraged differed for some of the professional characteristics, contextual factors, or information-seeking behaviors, but no consistent pattern in associations emerged.

**Conclusions:**

According to nursing staff, household activities are not as often encouraged as ADL or miscellaneous activities. Professional characteristics, contextual factors, and information-seeking behaviors are not consistently associated with the encouragement of functional activity. Nursing staff should also focus on improving the encouragement of household activities. Future research could examine the role of other factors in encouraging functional activity, such as experienced barriers, and assess to what extent the perception of nursing staff corresponds with their actual behavior.

## Background

The importance of encouraging functional activity among nursing home residents is widely recognized. Research shows that being active and performing functional activities is associated with less anxiety [1], less disruptive behavior [1], higher self-esteem [2], and a higher quality of life [3] in nursing home residents. In the United States, federal regulations require the provision of care to maintain the highest level of function among nursing home residents [4]. In the Netherlands, the Health Care Inspectorate emphasizes that nursing homes need to provide care that stimulates activity among residents, encouraging them to be active and perform functional activities on their own, instead of nursing staff taking over activities [5]. Encouragement could take place during activities of daily living (ADL) or during household activities, but also, for example, by discussing with the residents themselves which activities they previously conducted and motivating them to keep on performing them.

Promoting functional activity among residents is not only an opportunity nursing staff have, but also an important part of their job. In the past, nursing homes were mainly organized according to a medical model [6], in which nursing staff focused on taking care of the physical needs of residents. Currently, the autonomy of residents is crucial and more nursing homes strive to provide homelike environments, in which nursing staff encourage residents to continue their previous activities, including functional ones, as much as possible [7]. In spite of this, research has shown that residents are largely inactive [8–10]. Residents’ participation in, for example, household activities is low [3, 10]. Nursing staff may be able to play a more substantial role in encouraging functional activities [10].

There is a lack of research regarding the extent to which nursing staff stimulate residents to be active. In addition, it is unknown how this encouraging behavior varies. Research regarding the use of evidence-based or best practices in nursing care indicates that different factors come into play [11–14], including professional characteristics of the nursing staff, such as age [11], educational level [12, 13], or years of professional experience [11]; and contextual circumstances, such as staff mix [15] or ward type [11, 12]. In addition, studies have shown that the information-seeking behavior (for example, reading professional journals) of nursing staff may be associated with the use of evidence-based practices [12, 14].

It is not known how professional characteristics, contextual factors, or information-seeking behaviors are associated with the extent to which nursing staff encourage functional activity among nursing home residents. Therefore, we have conducted a cross-sectional study with a twofold purpose: first, to examine the extent to which nursing staff in the Netherlands perceive that they encourage functional activity in nursing home residents; and, second, to examine the association between these perceptions and various professional characteristics, contextual factors, and information-seeking behaviors of nursing staff.

## Methods

### Context: Nursing homes in the Netherlands

In nursing homes in the Netherlands, a distinction is made between residents with chronic physical problems, who live in somatic wards, and residents with psychogeriatric problems, such as dementia, who live in psychogeriatric wards [16]. Dutch nursing homes provide more complex continuing care and monitoring compared with residential care homes [16]. The meals are often taken in the wards and in many nursing homes small kitchen facilities are available in the ward, for example to prepare breakfast. The majority of the workforce in Dutch nursing homes are certified nurse assistants (CNAs) who receive three years of secondary-vocational training. In addition, care is provided by vocationally-trained or bachelor-educated registered nurses (RNs) who receive four years of training. Nursing homes are primarily non-profit organizations that are united in Actiz, the Dutch organization of health care providers. In contrast to some other countries, in the Netherlands there are no national databases comprising detailed information on all nursing homes (such as resident characteristics, or the number or type of staff).

### Design and sample

**Fig. 1:**
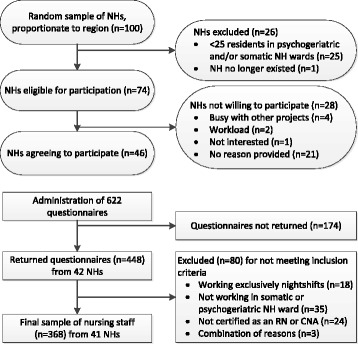
Flowchart of the study sample

A cross-sectional study was conducted among nursing staff of nursing homes in the Netherlands. From a list of nursing homes provided by Actiz, a random proportionate sample of 100 nursing homes was drawn by author NOK using the sampling procedure from IBM SPSS Statistics for Windows (Version 22.0. Armonk, NY: IBM Corp). Nursing homes were stratified according to five regions in the Netherlands (north, east, south, west, and central) and from each region a number of random nursing homes was drawn, proportionate to the total number of nursing homes in that region. Next, to warrant the exclusion of care homes with a single small nursing home ward, author NOK verified by telephone if the 100 selected nursing homes provided care to at least 25 somatic and/or 25 psychogeriatric nursing home residents. Twenty-five facilities were excluded because they did not meet this criterion and one nursing home no longer existed at the time of recruitment. Of the remaining 74 nursing homes, 46 agreed to participate (see Fig. 1 for a flowchart). In this sample, nursing homes from all regions were represented, they were distributed largely according to the proportionate sample that was drawn; 11% of the nursing homes were situated in the north, 9% in the east, 39% in the south, 37% in the west, and 4% in the central region of the Netherlands.

Based on practical considerations, nursing homes with both somatic and psychogeriatric wards were asked to administer 16 questionnaires among the nursing staff (eight from each ward type); nursing homes with only one of these ward types were asked to administer ten questionnaires among the nursing staff. In total, 622 questionnaires were administered.

Nursing staff were eligible if they were RNs or CNAs. Nursing staff working exclusively on night shifts and nursing staff who did not have a contract for at least 12 h per week were excluded from this study because of their limited opportunities to encourage functional activities.

### Data collection

In each participating nursing home a local contact person was asked to distribute the questionnaires among eligible nursing staff, these nursing staff did not have to work in the same ward. The contact persons would collect and return the anonymously-completed questionnaires to the research team within two weeks. If the research team had not received the questionnaires within three weeks, they would either telephone or send an email reminder to the contact person. All data were collected in January and February 2014.

### Measures

#### Encouragement of functional activities (dependent variables)

The MAastrIcht Nurses Activity INventory-behaviors (MAINtAIN-behaviors) questionnaire [17] was used to measure the extent to which nursing staff perceive that they encourage residents to perform functional activities. The MAINtAIN-behaviors was developed using a comprehensive method in which its usability and content validity were established in a study involving experts, nursing staff, residents, and other nursing home professionals [17]. The MAINtAIN-behaviors comprises three subscales assessing the degree to which nursing staff perceive they encourage residents to perform various types of activities: first, an 8-item subscale for encouraging ADL, for example, encouraging independent bathing or showering; second, a 6-item subscale for encouraging household activities, such as encouraging setting and clearing the table; third, a 5-item subscale for miscellaneous encouraging activities, such as promoting participation in organized activities, discussing and maintaining previous activities, encouraging informal caregivers not to take over activities, discussing the residents’ preferred activities, and encouraging activity as part of the residents’ care plan. For each item of the MAINtAIN-behaviors, respondents could rate to what extent a certain activity was encouraged in their ward (“in my ward, we encourage…”). Answer options ranged from ‘1 = never’ to ‘9 = always’. Internal consistency for the subscales, using Cronbach’s alpha, in the present study was 0.83 for the ADL subscale, 0.79 for the household activities subscale, and 0.77 for the miscellaneous activities subscale.

#### Professional characteristics, contextual factors, and information-seeking behaviors (independent variables)

Based on literature several professional characteristics, contextual factors, and information-seeking behaviors were selected [11–15]. The professional characteristics comprised gender, age (≤35 years, >35 years ≤50, >50 years), profession (CNA or RN), years of professional experience in the care for older persons (≤10 years, >10 years ≤20, >20 years), and number of work hours per week (≥12 h per week ≤26, >26 h per week <32, ≥32 h per week). The contextual factors consisted of ward type (psychogeriatric or somatic), and staff mix (proportion of RNs in the ward, i.e., the number of RNs divided by the total number of RNs and CNAs that worked in the ward, according to the respondent).

The information-seeking behaviors included how often respondents referred to specific information sources on care problems (websites, Dutch professional journals, English-language journals, guidelines, colleagues, and experts) or how often they attended specific activities to keep their professional skills and knowledge up-to-date (conferences, courses within their organization, courses outside of their organization, clinical courses in the ward, and reading groups). The behaviors were assessed using single-item questions that were developed for this study. First, it was assessed how often respondents used specific information sources in the past three months. Second, respondents indicated how often they attended professional development activities in the past 12 months. After recoding, the answer categories for each source of information or activity comprised ‘never’ or ‘≥1 time’ in the past three or 12 months.

### Statistical analyses

Descriptive statistics were used to determine percentages for the categorical variables. Mean scores and standard deviations were determined for each subscale of the MAINtAIN-behaviors. For each subscale, missing values on the items were imputed with the respondent’s average score for the other items, if at least 75% of the items of that subscale had been completed. Missing values for the ADL, household, and miscellaneous subscales were imputed for a total of 4.9, 2.4, and 1.9% of the respondents, respectively.

Mean scores of the three subscales were compared by conducting paired-samples t-tests, with a Bonferroni correction to account for multiple testing. Additional analyses were performed to provide an overview of the extent to which respondents encouraged activity among residents. For these analyses, the answer options of the MAINtAIN-behaviors items were categorized into ‘(almost) never’ (score 1–3), ‘sometimes’ (score 4–6), and ‘(almost) always’ (score 7–9).

For each independent variable (professional characteristics, contextual factors, and information-seeking behaviors) mean scores and standard deviations of the three MAINtAIN-behaviors subscales were calculated. Hierarchical linear regression analyses (random intercept) were performed to determine the association between each independent variable and each subscale of the MAINtAIN-behaviors (possible range 1–9). In each model one independent variable was used, no additional variables were added to these models. In order to account for the hierarchical structure of the data, nursing staff (level one) were grouped by nursing home (level two). For all models, estimated marginal means, standard errors, *p*-values, and intra-class coefficients (ICCs) were determined. For the independent variables with three categories (i.e., age, professional experience, and work hours per week), each category was used as a reference for the other two categories in the analyses (i.e., the first category was compared with the second category, the second category was compared with the last category and the last category was compared with the first category). *P*-values <0.05 were considered statistically significant. Sensitivity analyses were conducted by imputing missing values on the items of the ADL, household, and miscellaneous subscales with 1 and with 9 instead of the respondent’s average score of the other items within that scale. All statistical analyses were performed using IBM SPSS Statistics for Windows (Version 22.0. Armonk, NY: IBM Corp).

## Results

### Sample characteristics

A total of 448 respondents from 42 nursing homes completed the MAINtAIN-behaviors (response rate 72%; range per nursing home 50–100%), but 80 questionnaires had to be excluded, because the respondents did not meet the inclusion criteria (see Fig. 1). The 368 eligible respondents represented 41 nursing homes (sample characteristics are displayed in Table 1); 275 (75%) of them were CNAs and 231 (63%) worked in a psychogeriatric ward. Information-seeking behaviors varied, e.g., 5% had searched for information in an English-language journal in the past three months, whereas 99% had consulted a colleague.

**Table 1:** Sample characteristics (*N* = 368)^a^

	Number (%)
Professional characteristics	
Gender	
Female	346 (94)
Age	
≤ 35 years	116 (33)
> 35 years ≤50	141 (40)
> 50 years	95 (27)
Profession/educational level	
CNA	275 (75)
RN	93 (25)
Professional experience	
≤ 10 years	127 (38)
> 10 years ≤20	100 (30)
> 20 years	108 (32)
Work hours per week	
≥ 12 h per week ≤ 26	109 (30)
> 26 h per week < 32	83 (23)
≥ 32 h per week	169 (47)
Contextual factors	
Ward type	
Psychogeriatric ward	231 (63)
Somatic ward	137 (37)
Staff mix: proportion of RNs in the ward	
≤ 0.11	153 (49)
> 0.11	160 (51)
Information-seeking behaviors	
≥ *1x past three months*	
Reading on websites	68 (19)
Reading Dutch professional journals	171 (49)
Reading English-language journals	16 (5)
Reading guidelines	343 (96)
Consulting a colleague	358 (99)
Consulting an expert	276 (75)
≥ *1x past year*	
Attending a conference	112 (31)
Attending a course within the organization	332 (91)
Attending a course outside the organization	133 (38)
Participating in a clinical course in the ward	254 (70)
Participating in a reading group regarding care	15 (4)

*CNA* Certified nurse assistant, *RN* Vocationally-trained or bachelor-educated registered nurse

^a^ *N* does not always add up to 368 due to missing data

### Encouragement of functional activities

Table 2 displays the perceived encouragement of functional activities. The mean scores for the ADL subscale, the household activities subscale, and the miscellaneous activities subscale were 6.9 (SD 1.2), 4.1 (SD 1.9), and 6.7 (SD 1.5), respectively, out of a theoretical range from 1 to 9. These mean subscale scores differed significantly from each other (*p* < 0.001 for all comparisons after Bonferroni correction). More than half of the respondents (66–86%) stated that ADL were (almost) always encouraged, but the need for assistive devices for independent dressing was not always discussed (41%). Less than half of the respondents reported that household activities were (almost) always encouraged (ranging from 11% for folding or putting away clothes to 45% for preparing sandwiches). Regarding miscellaneous encouraging activities, the majority of the respondents (50–83%) indicated that all activities were (almost) always performed. For example, according to 83% of the respondents, residents were (almost) always encouraged to participate in organized activities, such as wheelchair dancing.

**Table 2:** Perceived encouragement of ADL, household activities, and miscellaneous activities: means and item-scores per subscale (*N* = 368^a^)

Subscales		Mean [SD]	
ADL		6.9 [1.2]	
Household activities		4.1 [1.9]	
Miscellaneous activities		6.7 [1.5]	
	(Almost) never N (%)	Sometimes N (%)	(Almost) always N (%)
Items ADL subscale			
Closely follow independent ADL performance	14 (4)	109 (30)	243 (66)
Encourage independent performance of ADLs	11 (3)	92 (25)	263 (72)
Discuss assistive devices for eating	31 (8)	93 (25)	242 (66)
Compliment residents on dressing and undressing	11 (3)	58 (16)	297 (81)
Discuss assistive devices for independent dressing	89 (24)	126 (34)	151 (41)
Closely follow independent movement	1 (<1)	61 (17)	304 (83)
Encourage independent movement	6 (2)	46 (13)	314 (86)
Provide assistive devices for bathing	25 (7)	89 (24)	252 (70)
Items household activities subscale			
Prepare sandwiches	51 (14)	149 (41)	166 (45)
Encourage setting and clearing the table	89 (24)	120 (33)	157 (43)
Make the beds	204 (56)	94 (26)	68 (19)
Encourage folding or putting away clothes	222 (61)	104 (28)	39 (11)
Encourage light household activities	175 (48)	108 (30)	82 (22)
Discuss with residents household chores they can help with	166 (45)	116 (32)	84 (23)
Items miscellaneous activities subscale			
Encourage participation in organized activities	8 (2)	56 (15)	302 (83)
Discuss and maintain the residents’ previous activities	23 (6)	100 (27)	242 (66)
Encourage family/informal caregivers to only help residents when they cannot do something themselves	47 (13)	135 (37)	183 (50)
Encouraging physical activity is part of care plan	35 (10)	98 (27)	233 (64)
Discuss preferred activities	60 (16)	105 (29)	201 (55)

Mean subscale scores are calculated based on the means of the original 9-point scale scores of all the items within that subscale; the scores can range from 1 (never encouraged) to 9 (always encouraged)

*ADL* activities of daily living

^a^N does not always add up to 368 due to missing data. Answers scored on the 9-point scale were categorized into ‘(almost) never’ (scores 1-2-3), ‘sometimes’ (4-5-6) and ‘(almost) always’ (7-8-9)

### Factors associated with the perceived encouragement of functional activity

Table 3 shows the unadjusted mean scores for the ADL activities subscale, for the household activities subscale, and for the miscellaneous activities subscale per professional characteristic and contextual factor. These scores are similar to the estimated marginal means that resulted from the hierarchical linear regression analyses, therefore, only the unadjusted means are presented. The largest difference in the perceived encouragement of activities, in particular household activities, was between respondents working in different ward types. The hierarchical linear regression analyses showed that respondents working in psychogeriatric wards reported significantly more often that household activities were encouraged compared with respondents working in somatic wards (*p* < 0.001, mean score 4.8, SD 1.6 and 3.7, SD 1.6, respectively). The perceived encouragement of miscellaneous activities also differed significantly between respondents from psychogeriatric and from somatic wards, but the difference was smaller (*p* = 0.001, mean score 6.9, SD 1.4 and 6.4, SD 1.4, respectively). As Table 3 shows, the only other professional characteristics or contextual factors significantly associated with the encouragement of activities were age and work hours per week (associated with the subscale of miscellaneous activities).

**Table 3:** Mean encouragement of ADL, household activities and miscellaneous activities per professional characteristic and contextual factor

	ADL subscale		Household activities subscale		Miscellaneous activities subscale	
	Mean^a^	SD	Mean^a^	SD	Mean^a^	SD
Professional characteristics						
Gender						
Male	6.9	(1.2)	4.5	(1.8)	6.6	(1.3)
Female	7.0	(1.2)	4.4	(1.7)	6.7	(1.5)
Age^b^						
≤ 35 years	6.9	(1.2)	4.2	(1.6)	6.5^c^	(1.5)
> 35 years ≤50	7.1	(1.1)	4.6	(1.7)	6.9^c^	(1.3)
> 50 years	7.1	(1.3)	4.4	(1.8)	6.7	(1.6)
Profession/educational level						
CNA	7.1	(1.2)	4.4	(1.7)	6.8	(1.5)
RN	6.9	(1.2)	4.4	(1.8)	6.5	(1.4)
Professional experience^b^						
≤ 10 years	7.0	(1.2)	4.3	(1.7)	6.7	(1.5)
> 10 years ≤20	6.9	(1.2)	4.3	(1.7)	6.6	(1.6)
> 20 years	7.1	(1.2)	4.6	(1.8)	6.8	(1.4)
Work hours per week^b^						
≥ 12 h per week ≤26	7.0	(1.5)	4.3	(1.5)	6.6	(1.4)
> 26 h per week < 32	7.0	(1.0)	4.5	(1.7)	6.5^d^	(1.5)
≥ 32 h per week	7.0	(1.3)	4.4	(1.9)	6.9^d^	(1.4)
Contextual factors						
Ward type						
Psychogeriatric ward	7.1	(1.2)	4.8^e^	1.6	6.9^e^	(1.4)
Somatic ward	6.9	(1.2)	3.7^e^	1.6	6.4^e^	(1.4)
Staff mix: proportion of nurses in the ward						
≤ 0.11	7.0	(1.2)	4.4	(1.7)	6.8	(1.5)
> 11	7.0	(1.2)	4.4	(1.8)	6.6	(1.4)

*CNA* Certified nurse assistant, *RN* Vocationally-trained or bachelor-educated registered nurse

^a^Unadjusted means are presented, these are similar to the estimated marginal means resulting from the hierarchical linear regression analyses (random intercept; level 1 - nursing staff, level 2 - nursing home) between each independent variable and each subscale of the MAINtAIN-behaviors (range 1–9). Indicated statistical significant differences (*p* < 0.05) are based on these analyses. No additional variables were added to the models. ICCs range from 0.06-0.10, 0.16-0.19 and 0.02-0.05 for the models with the outcome measure ADL, household activities and miscellaneous activities, respectively

^b^For variables with three categories, each category was used as a reference for the other two. Because of these variables and to increase the comprehensibility of the table, no *p*-values are presented

^c^Statistical significant differences between age ‘≤35 years’ and ‘>35 years ≤50’

^d^Statistical significant difference between working ‘>26 h per week < 32’ and ‘≥32 h per week’

^e^Statistical significant difference between psychogeriatric ward and somatic ward

Due to missing data, sample size for each analysis varies from 311 to 366

Table 4 presents the unadjusted mean scores for the functional activity subscales for each information-seeking behavior. Again, these mean scores were similar to the estimated marginal means resulting from the hierarchical linear regression analyses. On the whole, few of the information-seeking behaviors were significantly associated with the encouragement of functional activities, most of the associations found were with the encouragement of household activities. The hierarchical linear regression analyses revealed that respondents who searched on websites, attended conferences, participated in clinical courses in the ward, or in reading groups regarding care reported significantly more encouragement of household activities in their wards.

**Table 4:** Encouragement of ADL, household activities and miscellaneous activities: means and associations per information seeking - behavior

	ADL subscale			Household activities subscale			Miscellaneous activities subscale		
	Mean^a^	SD	*P* value	Mean^a^	SD	*P* value	Mean^a^	SD	*P* value
Reading on websites									
< 1× past 3 months	6.8*	(1.3)		4.0*	(1.6)		6.6	(1.6)	
≥ 1× past 3 months	7.1*	(1.2)	0.026	4.5*	(1.7)	0.004	6.7	(1.4)	0.479
Reading Dutch professional journals									
< 1× past 3 months	6.9	(1.2)		4.2	(1.7)		6.7	(1.4)	
≥ 1× past 3 months	7.1	(1.2)	0.212	4.5	(1.7)	0.072	6.8	(1.4)	0.587
Reading English-language journals									
< 1× past 3 months	7.0	(1.2)		4.3	(1.7)		6.7	(1.4)	
≥ 1× past 3 months	6.6	(1.4)	0.360	4.7	(2.1)	0.290	6.8	(1.6)	0.831
Reading guidelines									
< 1× past 3 months	6.7	(0.8)		4.2	(1.6)		6.4	(1.3)	
≥ 1× past 3 months	7.0	(1.2)	0.325	4.4	(1.7)	0.604	6.8	(1.4)	0.274
Consulting a colleague									
< 1× past 3 months	6.8	(0.8)		4.4	(1.5)		5.6	(2.3)	
≥ 1× past 3 months	7.0	(1.2)	0.515	4.4	(1.7)	0.453	6.7	(1.4)	0.057
Consulting an expert									
< 1× past 3 months	7.0	(1.2)		4.4	(1.7)		6.6	(1.4)	
≥ 1× past 3 months	7.0	(1.2)	0.633	4.4	(1.7)	0.862	6.8	(1.4)	0.181
Attending a conference									
< 1× past year	7.0	(1.2)		4.3*	(1.7)		6.7	(1.4)	
≥ 1× past year	7.0	(1.2)	0.995	4.6*	(1.8)	0.028	6.8	(1.6)	0.274
Attending a course within the organization									
< 1× past year	7.1	(1.4)		4.4	(1.9)		6.7	(1.4)	
≥ 1× past year	7.0	(1.2)	0.621	4.4	(1.7)	0.938	6.7	(1.5)	0.909
Attending a course outside the organization									
< 1× past year	7.1	(1.1)		4.4	(1.7)		6.7	(1.5)	
≥ 1× past year	6.8	(1.3)	0.051	4.4	(1.8)	0.556	6.7	(1.5)	0.980
Participating in a clinical course in the ward									
< 1× past year	6.8*	(1.2)		4.2*	(1.7)		6.6	(1.6)	
≥ 1× past year	7.1*	(1.2)	0.009	4.5*	(1.7)	0.037	6.8	(1.4)	0.183
Participating in a reading group regarding care									
< 1× past year	7.0	(1.2)		4.3*	(1.7)		6.7*	(1.5)	
≥ 1× past year	7.4	(0.9)	0.216	5.7*	(1.4)	0.004	7.7*	(1.1)	0.008

^a^Unadjusted means are presented, these are similar to the estimated marginal means resulting from the hierarchical linear regression analyses

^*^Statistically significant difference (*p* < 0.05). Associations between each information-seeking behavior and each subscale of the MAINtAIN-behaviors (range 1-9) were determined using hierarchical linear regression analyses (random intercept; level 1 - nursing staff, level 2 - nursing home), *p*-values presented are based on these analyses. No additional variables were added to the models. ICCs range from 0.06-0.09, 0.16-0.19 and 0.03-0.05 for the models with the outcome measure ADL, household activities and miscellaneous activities, respectively. Due to missing data, sample size for each analysis varies from 314 to 364

Sensitivity analyses in which missing values on the functional activity subscales were imputed with either ‘one’ or ‘nine’ showed similar results for the analyses with the professional characteristics and contextual factors, as well as for the analyses with the information-seeking behaviors.

## Discussion

This study showed that, according to nursing staff, household activities are not as often encouraged among residents as ADL or miscellaneous activities are. Some professional characteristics, contextual factors, and information-seeking behaviors were associated with the perceived encouragement of functional activity. However, no consistent pattern has emerged.

Although no previous research has analyzed the extent to which functional activities are encouraged by nursing staff, there are studies looking into the behavior of residents. These show that residents are largely inactive and rarely participate in household activities [3, 10]. For example, in an observation study among residents of seven nursing homes in the south of the Netherlands, Den Ouden et al. (2015) showed that residents were engaged in household activities, but only in less than 3% of their observations. This percentage is lower than one would expect, given the results of the present study, in which 39 to 86% of the nursing staff stated that certain household activities were encouraged - at least sometimes. The differences between the two studies are quite large and may be explained by the different concepts measured, i.e., perceptions of nursing staff versus behavior by residents, and by the difference in the respective samples, i.e., randomly selected nursing homes throughout the Netherlands versus a convenience sample of nursing homes in the south of the Netherlands. Another explanation may be that encouragement by nursing staff does not always result in increased functional activity among residents.

The finding that household activities were not often encouraged, compared with ADL, or miscellaneous activities, contrasts with the culture change [7] that is currently taking place in many nursing homes across the world. Nowadays, many nursing homes strive to maintain the meaningful activities residents previously conducted, including household activities [18–20]. In some nursing homes, it is expected that nursing staff prepare dinner together with residents [18]. In the Netherlands, this care philosophy particularly occurs in (small-scale) psychogeriatric wards [18]. Indeed, in the present study, nursing staff from psychogeriatric wards stated significantly more often that household activities were encouraged, compared with nursing staff from somatic wards.

In this study, few associations were found between professional characteristics, contextual factors, and information-seeking behaviors, and the perception whether or not functional activities were encouraged. Associations that were found were inconsistent; factors that were associated with the perceived encouragement of household activities were not associated with the perceived encouragement of ADL. Furthermore, factors that were significantly associated with this perceived encouragement reflected relatively small differences. The different kinds of measures that were used in this study may explain the inconsistent findings. Encouraging functional activities refers to specific behaviors (i.e., specific daily activities), while the information-seeking behaviors were measured on a more general level. For example, respondents were asked if they attended courses in general, they were not asked if they attended courses focused on the encouragement of functional activities. Searching for information in a specific area does not necessarily imply knowledgeability about the encouragement of functional activities. In addition, although we carefully selected our independent variables drawing on previous studies [11–15], it might be that the professional characteristics, contextual factors, or information-seeking behaviors used are not the most important factors for encouraging functional activity. Perhaps more closely-related factors, specific barriers or facilitators, such as the perceived capabilities of residents, support of colleagues [17, 21], or the availability of domestic facilities in the wards determine whether or not functional activities are encouraged.

The findings of this study indicate that nursing staff prefer sources that allow interaction, such as colleagues or clinical lessons, over traditional sources of knowledge, such as journals. This is in accordance with previous research investigating the knowledge sources of nursing staff [22]. Given the relatively low educational level of nursing staff in nursing homes, it is not surprising that only very few nurses in this study actively searched for written information. However, to warrant the quality of care in nursing homes, it is important that evidence-based or best practices reach the nursing staff. To ensure that nursing staff encourage functional activity, nursing homes need to use strategies that meet the preferences and competences of their nursing staff. Changing nursing behavior may be best done by using interactive strategies. To achieve this, it is essential that people with the appropriate knowledge and skills are available in the nursing home.

For this study, a proportionate random sample of nursing homes was drawn from different regions within the Netherlands, resulting in a, from a national perspective, large sample representing nursing home staff throughout the country. This is one of the few studies in the Netherlands that involved so many nursing homes. Most of the nursing homes that agreed to participate in this study were situated in the south of the Netherlands and least in the central region; their distribution was largely similar to the proportionate random sample that was drawn. The results of this study can be used as a reference for other researchers or nursing homes that want to use the MAINtAIN-behaviors to measure the extent to which nursing staff perceive to encourage functional activities. However, the population in the present study might not be entirely representative for nursing home staff in other countries. The majority of the nursing staff participating in this study were CNAs, who are comparable to the licensed practical nurses in the United States [23]. Dutch CNAs receive a three-year secondary-vocational training. In contrast, for example, in the United States the majority of the nursing home staff are nursing assistants who receive a minimal training of 75 h [24, 25].

### Limitations

The present study has a cross-sectional design; therefore, no causal relationships could be assessed. In addition, the aim of this study was to assess the extent to which nursing staff perceive that they encourage functional activity; therefore it assessed nursing staff perceptions, which may not necessarily be the same as the extent to which they actually encourage functional activity. Furthermore, respondents were asked to reflect upon their ward (“in my ward, we encourage…”), which might not always completely correspond to their own personal behavior. For a more objective perspective, observations could be conducted regarding the extent to which nursing staff encourage functional activities. Moreover, other contextual factors, such as the availability of specific domestic facilities, could also have been included in this study.

### Implications for research and practice

The present study examined the association between professional characteristics, contextual factors, and information-seeking behaviors, and the perceived encouragement of functional activity. Future studies could consider factors that are possibly more closely-linked to the encouragement of functional activities, for example specific barriers or facilitators nursing staff perceive towards encouraging functional activity (e.g., capabilities of residents, self-efficacy of nursing staff, support of colleagues, or time constrains [17, 26, 27]). Furthermore, future studies could examine how the perception of nursing staff corresponds with their actual behavior, and if increased encouragement by nursing staff leads to improved functional activity among residents.

This study showed that household activities were less often encouraged than other activities, according to the nursing staff. Performing household activities is associated with a higher quality of life among nursing home residents [3]. Here lies an opportunity for nursing homes; nursing homes could focus on improving the extent to which household activities are encouraged and nursing staff should be aware of the importance of these kinds of activities.

## Conclusion

The findings of this study show that, according to the nursing staff, most household activities are not often encouraged by a large proportion of the nursing home staff. ADL and miscellaneous activities are more often perceived to be encouraged. Professional characteristics, contextual factors, and information-seeking behaviors are not consistently associated with the encouragement of functional activity. Future studies aimed at improving the encouragement of functional activity could focus on the encouragement of household activities, the association between perceptions and actual behavior of nursing staff, and potential barriers and facilitators for encouraging residents to participate in functional activities. Furthermore, studies providing insight into whether or not encouragement of functional activity by nursing staff leads to improved functional activity among nursing home residents are necessary.

## Abbreviations

ADL: Activities of Daily Living
CNA: Certified nurse assistant
MAINtAIN: MAastrIcht Nurses Activity INventory
RN: Vocationally-trained or bachelor-educated registered nurse
